# Non-traumatic dislocation (Cam Jump) in a revision knee: a case report

**DOI:** 10.1186/1757-1626-2-7001

**Published:** 2009-04-28

**Authors:** Buchi Rajendra Babu Arumilli, Bernard Ferns, Martin Smith, Ramesh Thalava, Elmoez Obeid, Bishalahalli Muddu

**Affiliations:** Department of Trauma & Orthopaedics, Tameside General HospitalFountain street, Ashton-Under-Lyne, OL6 9RWUnited Kingdom

## Abstract

Dislocation after total knee arthroplasty is a difficult problem and is even more challenging if it occurs following revision. We report the case of a 82 year old male presenting after a frank posterior dislocation (Cam Jump) in a posterior stabilized revision knee arthroplasty without trauma. Flexion space instability with extensor insufficiency was presumed to be the cause of the dislocation without significant trauma. The possibility of worsening collateral stability with high flexion ranges following knee replacement is also explored.

## Introduction

Dislocation after Total knee arthroplasty is an uncommon and a difficult problem to address, more so if this happens after a revision. Instability is the third most common cause of failure of a total knee arthroplasty [1]. Tibio-femoral instability is caused mainly by ligamentous imbalance, malalignment, and implant wear or fixation failure [2]. In the literature, there are reports of dislocation in total condylar [3], posterior stabilized [2] and constrained knee systems [4]. The posterior stabilized knee design claimed advantages like increased flexion, better tissue balancing and better polyethylene wear characteristics [5]. Dislocations in primary posterior stabilized knees (Insall-Burstein II [5,6,7] and Kinemax models [2,8]) have been reported with the eponymous “Cam Jump” due to different reasons. In revision arthroplasties, reports of such dislocations are few. Sharkey et al [9] described seven cases of dislocation after knee Arthroplasty which included one patient with a posterior stabilized total condylar revision knee. We present an uncommon case of a frank posterior dislocation in a posterior super stabilized Kinemax revision knee replacement (Howmedica, Rutherford, New Jersey) which occurred without trauma, 3 years after revision along with a brief review of relevant literature and speculate the cause of this relatively rare situation.

## Case presentation

A 74-year-old Caucasian male underwent a primary total knee replacement (Kinemax cruciate sacrificing) on the left side for osteoarthritis. He was a retired pharmacist. No other co-morbidities or a significant family history was present. Pre-operatively there was a 10 degree varus deformity of left knee and x-rays showed gross tricompartmental osteoarthritis with total loss of medial joint space.

He sustained a traumatic dislocation of the left knee 22 years ago, which was managed by closed reduction under general anesthesia followed by cast immobilization and physiotherapy. Examination under anesthesia of the knee after reduction following the traumatic dislocation showed anteromedial instability for which a long leg cast was given for 8 weeks. He did not have any following symptomatic instability.

**Figure 1.**
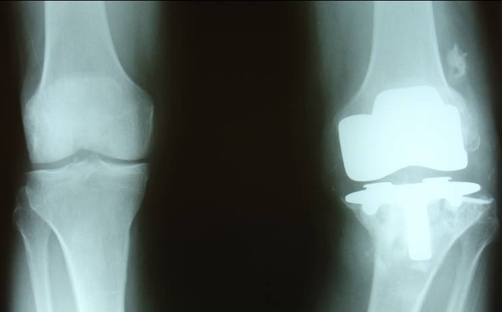
AP x-ray after 5 years of primary left knee arthroplasty showing premature aseptic failure.

Intra-operatively during the primary surgery the posterior cruciate was absent, the flexion and extension balancing was thought to be adequate after using a 15 mm tensioning device. There was no evidence of a collateral insufficiency. The post operative period was uneventful. He had premature aseptic loosening (Figure 1) of the tibial tray at five years after the index procedure for which a revision was performed. Infection was ruled out as the cause of this early failure. During revision there was antero-medial tibial bone loss that was addressed with bone graft and a 5 mm augment. The revision was performed using a Kinemax Plus super-stabilizer knee system (Howmedica, Rutherford, New Jersey) using a large femoral, a large tibial implant and a 12 mm insert with distal femoral augmentation. He had 100 degree of flexion in the immediate post-operative period which improved to 120 degrees in the subsequent follow-ups. The last follow-up was at 12 months post revision when he had 0-120 degrees of flexion and no pain.

**Figure 2.**
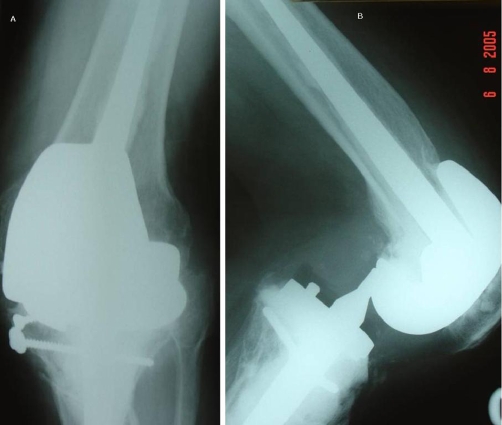
**(A)** AP x-ray of the revision knee arthroplasty at dislocation. **(B)** Lateral x-ray of the revision knee arthroplasty at dislocation (showing the “Cam jump”).

**Figure 3.**
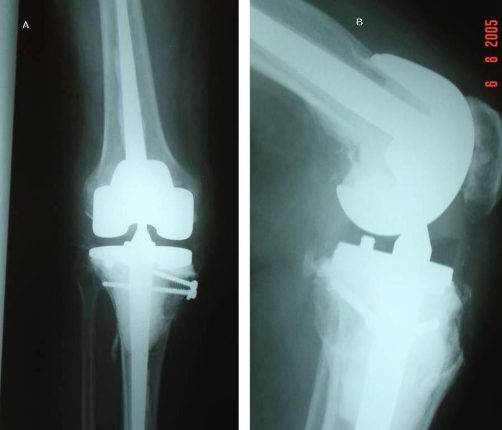
**(A)** AP of the revision arthroplasty after reduction. **(B)** Lateral of the revision arthroplasty after reduction.

He was subsequently admitted with obsessive compulsive disorder to a psychiatric ward. He was referred with a painful locked left knee without any history of trauma (36 months post revision surgery). On clinical examination the left knee was painful and locked in 60 degree flexion. The patient denied any history of trauma or twisting episode and stated that his knee “gave way” while getting up from a chair. The x-rays showed the left knee in flexion and a posterior dislocation of the knee with the femoral Cam totally anterior to the tibial support post, without significant deformity in the coronal plane (Figures 2A & 2B). Closed reduction was obtained, with the knee in flexion and by applying anterior pull on the distal femur and traction along the axis of the tibia (Figures 3A & 3B). After reduction, EUA showed only mild instability in extension, but in flexion it was significantly unstable showing anterior translation (Drawer's) of tibia. He was put on a long leg cast for 6 weeks and later the knee was mobilized in a hinged knee brace restricting flexion up to 70 degrees which prevented any subsequent episodes of dislocation.

## Discussion

Dislocation of a knee after total knee replacement is being increasingly reported in the literature. Knee dislocation after Total knee replacement was first reported in 1979 after total condylar knee replacement in 4 patients by Insall et al [3], in a series of 220 patients. The authors attributed the dislocations to inadequate stability in flexion which was addressed with revision to a thicker tibial insert. Since this first report by Insall et al, cases of dislocation in knee arthroplasties are being reported for various reasons. Table-1 lists such case reports of dislocation with the details of the type of prosthesis, number of patients and the year of reporting. This complication has been reported with every prosthesis design reinforcing the fact that the more important factors in deciding the long term result after a total knee is the technique and decision making rather than the design of the prosthesis itself.

**Table 1:** Case reports of dislocations after total Knee arthroplasty in the literature (till 2004)

Author	Year	Type of Prosthesis	Primary/Revision	Number	Management
Insall et al	1979	Total condylar	Primary	4	Revision with a thicker tibial insert
Bargren	1980	Total condylar	Primary	1	Revision of tibial component
Den Hartog et al	1987	Semi constrained PCL retaining	Primary	1	
Goldberg et al	1988	Total condylar	Primary	-	
Hanssen & Rand et al	1988	Kinemax	Revision	1	MUA
Galinat et al	1988	Posterior stablised	Primary	2	2 MUA
Gebhard et al	1990	kinematic II stabiliser	Primary	2	1 MUA,1 revision of tibial component
Sharkey et al	1992	6 cruciate substituiting, 1 cruciate sparing	5 Primary 2 Revision	7	2 MUA, 1 cylinder cast, 1 patellar realignment, 3 component revision
Lombardi et al	1993	IB I-4, IBII-10, IBIImod-1	Primary	15	Not specified
Mills et al	1994	IB II	Primary	2	2MUA
Ochsner et al	1996	kinemax	Primary	2	2 MUA
Wang et al	1997	-	Primary	6	-
Erceg et al	2000	Posterior stabilised	Primary	1	1-revision
Gidwani et al	2001	Posterior stabilised	Primary	1	1-revision
Hossain et al	2001	IB II	Primary	3	1 insert change, 2 MUA
Huang et al	2002	LCS rotating platform	Primary	5	5-revision
Buechel	2003	LCS rotating platform	revision	2	2 revision of tibia
Chiu et al	2003	Posterior stabilised	Primary	1	1-revision
V Rao et al	2003	Mobile bearing prosthesis	Primary (multiple sclerosis)	1	Revision with a thicker insert
Tuoheti et al	2004	-	Primary	1	-
Thompson et al	2004	LCS rotating platform	Primary	10	9-open reduction,1- MUA

IB- Insall Burstein.

LCS- Low contact Stress.

MUA- Manipulation under anesthesia.

Boxes empty indicate that no details were available.

The posterior stabilized design was introduced to address the limitations of total condylar prosthesis, incorporating a femoral Cam and a tibial post to produce femoral roll back thereby increasing the possible range of flexion [10]. But there is a critical point beyond which an implant design allowing increasing flexion range would compromise knee stability [11]. Sharkey et al [9] in 1992 reported seven patients with knee replacement who had a posterior dislocation and postulated that extensor weakness along with flexion instability as the predominant cause of dislocation.

Kinemax knee system promoted a posterior stabilized design, which is quite popular. Gebhard et al [8] in 1990 reported two cases of dislocation in a primary Kinemax II stabilized arthroplasty. In both the patients a history of mediolateral stress in extreme flexion along with collateral ligament instability was present and the authors proposed that the anterior position (in comparison to Insall Burstein prosthesis) of the tibial post in the antero-posterior plane with its inability to provide sufficient mediolateral stability, as the cause of the dislocation. The other case series of primary Kinemax posterior stabilized arthroplasty dislocation was by Ochsner et al [2] in 1996. The authors concluded that the upsloping tibial support post of the Kinemax prosthesis was the cause of instability in flexion.

To optimize knee kinematics, proper alignment in the 3 planes with restoration of the joint line is critical. The most important factor influencing the probability of dislocation in a posterior stabilized knee arthroplasty is obtaining symmetric flexion and extension gaps. Different types of posterior stabilized implant has been developed to provide increased stability to account for ligament insufficiency but still incorporate a basic Cam post mechanism [11]. The posterior cruciate-substituting revision design, with it's extended post-Cam mechanism, compensates for flexion laxity only to some extent.

Instability after total knee arthroplasty can be classified into Anteroposterior or flexion space, varus/valgus or extension space and global instability [1]. Each type of instability has a different pathology and can be satisfactorily managed only by addressing the specific problem. In our patient, the knee probably had some flexion extension mismatch since the index surgery leading to premature aseptic loosening. During revision an implant (superstabilizer [12]) was used to address this instability, with a central tibial support post of 20 mm height in front of the femoral Cam. This choice of the revision implant was not totally appropriate in this patient as any mismatch if not correctable at surgery must be addressed with a hinged implant. When the patient flexes the knee to >70°, the overlap of the femoral Cam and tibial support also defined as the Dislocation safety factor [13] decreases. This decrease in the overlap along with subtle extensor dysfunction and persisting instability in flexion (Globally unstable knee) have caused the femoral Cam to ride over the tibial post leading to a frank posterior dislocation without major trauma or implant breakage.

On the basis of his general health and functional state, we have managed him with the next best option of a hinged brace limiting his knee flexion. The ideal choice in this patient if he was fully functional is to revise it to a linked prosthesis. Specifically, in revision knee arthroplasty, bone loss and destruction of landmarks make restoration of the joint line more challenging. This report highlights the importance of soft tissue balance during revision arthroplasty and if not achievable for any reason, should be addressed with a hinge.

Another interesting issue from this report is the question whether soft tissues can cope with high flexion rates after revision arthroplasty without developing instability in the long term. In a cadaveric study [14], the mean tibiofemoral force in a well balanced knee neared 50 N at full extension. The mean force between 15° and 75° of flexion in this series was 15.5 N (SD 9.6), before rising in an exponential manner to a peak at a mean of 175 N (SD 104) at 150° of flexion. Such forces on compromised soft tissues in a revised knee, especially at extreme ranges of flexion, might lead to chronic attenuation of ligaments resulting in progressive instability in subsequent years. Hence, there is a valid argument at least in a few patients, who are less demanding functionally, of compromising flexion (above 90-100 degrees) for a long term stable knee.
